# Feasibility and Compatibility of a Biomass Capsule System in Self-Healing Concrete

**DOI:** 10.3390/ma14040958

**Published:** 2021-02-18

**Authors:** Arkabrata Sinha, Qi Wang, Jianqiang Wei

**Affiliations:** Department of Civil and Environmental Engineering, Francis College of Engineering, University of Massachusetts Lowell, Lowell, MA 01854, USA; Arkabrata_Sinha@student.uml.edu (A.S.); Qi_Wang1@student.uml.edu (Q.W.)

**Keywords:** biomass, polylactic acid, capsule, self-healing concrete, degradation, compatibility

## Abstract

Cracking can facilitate deteriorations of concrete structures via various mechanisms by providing ingress pathways for moisture and aggressive chemicals. In contrast to conventional maintenance methods, self-healing is a promising strategy for achieving automatic crack repair without human intervention. However, in capsule-based self-healing concrete, the dilemma between capsules’ survivability and crack healing efficiency is still an unfathomed challenge. In this study, the feasibility of a novel property-switchable capsule system based on a sustainable biomass component, polylactic acid, is investigated. Capsules with different geometries and dimensions were studied focusing on the compatibility with concrete, including survivability during concrete mixing, influence on mortar and concrete properties, and property evolution of the capsules. The results indicate that the developed elliptical capsules can survive regular concrete mixing with a survival ratio of 95%. In concrete containing 5 vol.% of gravel-level capsules, the compressive strength was decreased by 13.5% after 90 days, while the tensile strength was increased by 4.8%. The incorporation of 2 vol.% of sand-level capsules did not impact the mortar strength. Degradation and switchable properties triggered by the alkaline matrix of cement were observed, revealing the potential of this novel biomass capsule system in achieving both high survivability and self-healing efficiency in concrete.

## 1. Introduction

The high brittleness and low ductility make concrete susceptible to cracking in the presence of mechanical loadings or environmental attacks. The formation of cracks in concrete makes it more permeable by providing pathways for the ingress of external moisture and aggressive chemicals and thereby making concrete vulnerable to a variety of deteriorations, such as corrosion of steel reinforcement [1,2], carbonation [3], sulfate attacks [4], frosting damage [5], alkali-silica reaction [6,7] or their combinations [8,9]. Without timely repair, lethal degradation or premature failure under these multiple aging mechanisms can occur in concrete structures. The conventional crack repairing approaches, e.g. epoxy resin injection [10,11,12] and electrodeposition [13,14], can only repair the cracks from the surface to a limited depth, and these approaches involve the use of heavy machinery, significant man-power and down-time for the concrete structure disrupting daily use of infrastructures. Furthermore, increased maintenance costs due to labor and materials consumption in these conventional techniques make it essential to develop new concrete crack repairing techniques for truly resilient infrastructures.

One of the novel techniques to achieve a substantial crack capacity of composite materials without human intervention is self-healing. Different self-healing approaches have been investigated in concrete materials, including; (i) autogenous healing based on filling of cracks by hydration of unhydrated cement particles [15,16] or carbonation of the hydration products in the presence of external moisture and carbon dioxide [15,17]; (ii) autonomous healing, which is primarily achieved by incorporating engineered healing agents such as supplementary cementitious materials [18], mineral admixtures [19], polymer agents or water glass [20]; and (iii) microbial healing achieved by direct incorporation of bacteria spores or encapsulation of bio-agents into concrete that can implement the conversion of hydrolysis of urea or calcium compound (Ca-lactate) [21,22,23,24,25]. In recent investigations, advances in triggering crack self-healing via different mechanisms, such as UV light [26], ultrasonic wave [27], and the coupling with internal curing [28], have been developed in this field. In addition to crack closure and recovery of mechanical properties, improvements of deterioration resistance again a variety of aging mechanisms in self-healing concrete have been increasingly studied. An innovative method employing polypropylene microfibers in conjunction with crystalline admixtures was developed by Munhoz et al. [29] for autogenous self-healing of cracks caused by the alkali-aggregate reaction. In a recent investigation by Xue et al. [30], crystalline admixture coupled with magnesium oxide expansive agent was employed for crack-healing via carbonation precipitation by conditioning concrete samples in a chloride-rich environment. Another study conducted by Yoo et al. showed the potential for self-healing in ultra-high performance concrete reinforced with steel microfibers via calcite precipitation in the cracks [31]. It is worth noting that, although significant efforts have been made on concrete self-healing, capsule-based self-healing is still the most effective and economical way to achieve robust crack repair. This is especially the case for concrete structures subjected to extreme mechanical and environmental conditions.

The encapsulation prevents the participation of the healing agents during the hydration period of cement. Some healing agents, like bacteria, need encapsulation to survive the harsh environment inside the concrete so that they can become active when the cracks are formed [32,33,34]. Another role of capsules is to ensure that the healing agents can be released in the presence of cracks. To achieve these roles, the capsules should be designed with sufficient strength to survive the concrete mixing and weak enough to ensure the cracks propagate through the capsules and release the healing agents. To date, glass [35,36,37,38,39,40], ceramic [41], expansive clays [34,42], natural fibers [43,44], perspex [38], paraffin [45,46], wax [47], silica [48,49], silica gel [50], diatomaceous earth [51], urea-formaldehyde [52,53,54,55] polymethyl methacrylate [54,55], low alkali cementitious materials [56], recycled brick aggregates [57], biocapsules (made of carbide slag fly ash and desulfurized gypsum) [58], sugar-coated expanded perlite [59] and porous concrete [60] have been investigated as capsule shell materials. In polymeric microcapsules, urea-formaldehyde, polyurethane, and melamine-formaldehyde are amongst the most commonly used polymeric encapsulation materials [61]. Although a variety of materials have been studied, the dilemma between the high survivability in concrete mixing and the easy rupture upon cracks has not been substantially solved. To address these challenges, in this study, a novel property-switchable biomass-based capsule system made of polylactic acid (PLA), which can degrade, facilitate easy rupture and release of healing agents triggered by the alkaline environment of concrete, is investigated.

**Figure 1:**
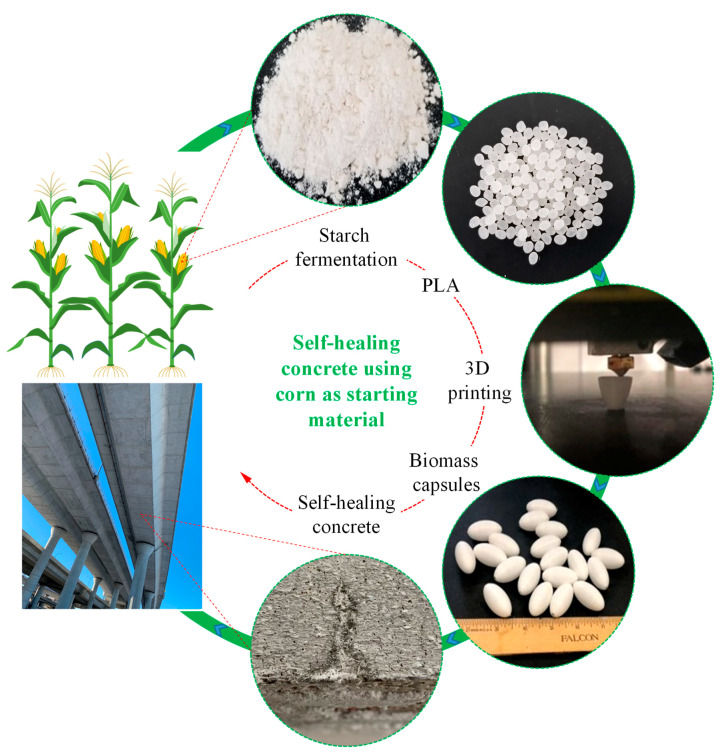
Schematic diagram of self-healing concrete using biodegradable corn as starting material.

As shown in Figure 1, PLA is a sustainable and biodegradable polymer synthesized from natural biomass resources such as corn, cassava, sugarcane, or sugar beet pulp [19,62]. It is a homopolymer or copolymer of L-lactic acid and/or D-lactic acid monomers, which are enantiomerically pure polymers that can be obtained from the polymerization of L-lactic acid and D-lactic acid, respectively [63]. As a biodegradable material, PLA is normally used in the biomedical industry as raw materials for anchors, screws plates, pins, rods, and other degradable components of medical implants [64]. Given its incredible printability and versatility, PLA has been widely used in the field of three-dimensional (3D) printing for the manufacturing of unique geometric models for bone regeneration [65], textiles [66], conductive components [67], and electrodes [68]. The hydrophobic nature, stiffness, tensile strength and gas permeability, comparable with polyethylene, polypropylene, polystyrene, and polyethylene terephthalate [69], make PLA an ideal shell material for encapsulation in self-healing concrete. The interfacial zone between PLA and the cement matrix indicates a favorable bonding thereby yielding an efficient breakage during concrete cracking while maintaining the mechanical properties of concrete [70]. Depending on the degree of crystallinity, molecular weight, and material morphology [71], PLA can exhibit a controlled rate of degradation. The hydrolytic degradation of PLA in neutral buffer phosphate solutions and the accelerated degradation at elevated temperatures of 37 °C [71,72], 50 °C, 70 °C, 97 °C [73] and 80 °C [74] and in alkaline 0.1 M [75] and 1.5 M NaOH solutions [76] have been investigated. According to Xu et al. [77], degradation of PLA can be significantly accelerated under elevated temperatures and in solutions with high pH values. This hydrolytic degradation behavior of PLA makes it possible to achieve switchable properties of capsules as a potential way to address the aforementioned challenges in self-healing concrete.

In this study, to leverage the physical and chemical features of PLA, capsules with different dimensions and geometry shapes were manufactured through 3D printing to simulate the sizes of coarse and fine aggregates used in concrete. The feasibility of using this novel concept of biomass capsules in self-healing concrete was evaluated by investigating the survivability of capsules in regular concrete mixing, the switchable properties of the capsules in the alkaline environment of concrete and mortar, as well as the influence of the capsules on the property development of mortar and concrete. The degradation behavior of the capsules was investigated through tension test, thermogravimetric (TGA), and microstructure analyses. The self-healing potential of the concrete containing the biomass capsules filled with sodium silicate was also preliminarily evaluated. 

## 2. Materials and Methods

### 2.1. Materials

PLA 4043D with an L-lactic/D-lactic ratio from 24:1 to 30:1, melting range from 150 °C to 180 °C (302–356 ℉) and a glass transition temperature from 55 °C to 60 °C (131–140 ℉), which is generally selected for 3D printing, was used for dog-bone specimens in a tensile strength test. Two PLA filaments, one white and one transparent, were used in 3D printing. The white filament (PLA^1^) was used for the manufacturing of gravel-size and sand-size capsules used in concrete and cement paste. The transparent filament (PLA^2^) was used to manufacture sand-size capsules in the matrix of mortar only. TGA analysis of unaged PLA shows that the onset and burnout temperature ranges are 320–356 °C and 374–443 °C, respectively. Reagent-grade sodium silicate (Na_2_SiO_3_) solution, with a composition of 10.6% NaO_2_ and 26.5% SiO_2_, was used as a healing agent. Type I/II Ordinary Portland cement, river sand, and all-purpose 3/8-inch gravels were used for mortar and concrete specimens. The specific gravity of the cement, fine aggregate, and coarse aggregate are 3.15, 2.7, and 2.6, respectively. The chemical and mineral compositions of the cement are summarized in Table 1. A high-efficiency poly carboxylate-based superplasticizer, ADVA® Cast 555, was used to improve the workability of concrete without segregation. Sodium hydroxide with a purity of over 97.0% was used to synthesize alkaline solutions.

### 2.2. Specimens

#### 2.2.1. PLA Dog-Bone Specimens for Alkaline Degradation Test

PLA dog bones with a gauge length of 100 mm, a gauge width of 12.5 mm and a thickness of 3.18 mm were prepared by using a Vista Edge injection molding machine (Lowell, MA, USA) through thermal injection. PLA degradation in the alkaline environment was investigated by immersing PLA dog bone specimens in a 0.1 M (mol/L) sodium hydroxide solution with a pH value of 13 under room temperature simulating the alkalinity of regular concrete pore solutions.

#### 2.2.2. Capsule Manufacturing

Hollow elliptical capsules were manufactured via fused deposition modeling (FDM) technique using a precision three-dimensional (3D) printer with a nozzle diameter of 0.4 mm. To obtain a smooth surface finish and accurate dimensions, the 3D printing was performed with an extruder temperature of 205 °C, a bed temperature of 60 °C, and a layer thickness of 0.2 mm at a print speed of 50 mm/s. In this study, the capsules were manufactured at two different size levels: gravel level and sand level. To investigate the influence of dimension and geometry on the survivability of the gravel-level capsules, 12 groups with two minor diameters of 9.5 mm and 19.05 mm simulating the commonly used gravel sizes (i.e. 3/8 inch and ¾ inch), different aspect ratios (major to minor diameter ratios of the ellipsoids) at 1:1:1, 1.5:1:1 and 2:1:1, and a series of shell thickness of 2.0 mm, 1.6 mm, 1.2 mm, 0.8 mm and 0.4 mm were studied. To investigate the compatibility of the biomass capsule in the matrix of mortar, sand-level capsules with a dimension of 7.5 × 5 × 5 mm^3^, an aspect ratio of 1.5, and a shell thickness of 0.4 mm were designed. The capsule-based healing system was developed by injecting sodium silicate (Na_2_SiO_3_) solution. The summary of the capsule dimension and geometry is presented in Table 2.

#### 2.2.3. Concrete Specimens

Concrete cylinders with a dimension of 76 mm by 152 mm, and 76 × 76 × 254 mm^3^ beams were cast based on a cement to sand to gravel ratio of 1:2:3. A water to cement ratio of 0.4 was employed and the water absorption of the aggregates was taken into account to adjust the amount of mixing water. A superplasticizer was used to improve the workability of concrete. A previous study by Lv and Chen [78] indicated that a capsule volume fraction between 2% and 5% can be selected by giving consideration to the minimum effect on concrete strength and the maximum self-healing capability. In this study, the volume fraction of capsules was fixed at 5% and 2% for the gravel-level capsules in concrete and the sand-level capsules in mortar specimens, respectively. The concrete matrix was mixed using a Gilson Brothers Co. concrete mixer according to ASTM C192 [79] followed by an additional 5 minutes of mixing after adding capsules. The slump test was performed according to ASTM C143 [80], and it was found that the slump of the concrete with PLA capsules is 0.5 in. (48%) higher compared to the plain concrete. After casting, the cylinder and beams specimens were covered to prevent water evaporation. The specimens were demolded after 24 h and cured in saturated lime water at room temperature (23 ± 2 °C) until testing.

#### 2.2.4. Mortar and Cement Paste Specimens

Mortar specimens with a dimension of 100 × 100 × 100 mm^3^ were prepared according to ASTM C109 [81] using Type I/II portland cement and river sand with a cement to sand ratio of 1:2.75 and a water to cement ratio of 0.40. Different from the concrete, the mortar specimens contain smaller sand-level capsules with a dimension of 7.5 × 5 × 5 mm^3^ at a volume fraction of 2%. 100 × 100 × 100 mm^3^ cement paste cubes were also prepared to investigate the influence of elevated temperature on the capsule’s degradation rate. For these cement paste samples, a cement ratio of 0.40 was employed and only two sand-size capsules were embedded. The cubes were covered with plastic wrap to prevent water evaporation followed by demolding after 24 h. After demolding, the mortar and cement paste cubes were cured in saturated lime water at room temperature (23 ± 2 °C) and 50 °C, respectively.

### 2.3. Experimental Procedure

#### 2.3.1. Tensile Test on Dogbone PLA Specimens

A tension test was conducted on the dogbone specimens to understand how PLA performs in a simulated service condition of the concrete matrix and to predict how the capsules rupture under tension. The tension test of the raw and aged PLA dog-bone specimens after being immersed in the 0.1 M sodium solution for 7 days, 14 days, 1 month and 3 months were performed on an Instron universal testing machine at a displacement rate of 5.08 mm/min according to ASTM D638 [82]. Three repetitions were employed for each testing age.

#### 2.3.2. Survivability Test

**Figure 2:**
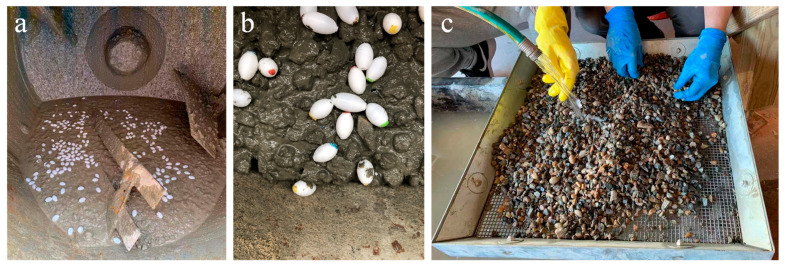
Survivability test procedure: (**a**) and (**b**) mixing, and (**c**) washing and capsule separation.

To determine the resistance of capsules to concrete mixing, a survivability test was performed by mixing the capsules with concrete in a revolving drum tilting mixer according to ASTM C192 [79]. After the standard concrete mixing procedure to ensure uniform mixing of the concrete components, the capsules were subsequently added and mixed for an additional 5 min (see Figure 2a,b). Twenty capsules from each group and a total of 12 groups were tested for survivability. A concrete quantity of 0.058 m^3^ was mixed to ensure a low capsule volume percentage of 0.82%. As shown in Figure 2c, after the mixing process, the fresh concrete was washed under running water to remove the cement binders and sieved to remove fine aggregates. The intact capsules from each group were collected for the calculation of intact ratios based on the number of intact capsules after mixing and the number of capsules mixed initially (i.e. 20 for each group). The intact capsules without leakage were considered active capsules. The survival ratio was calculated as the ratio of the active capsules to the number of capsules mixed initially for each group.

#### 2.3.3. Compressive and Split Tensile Tests

**Figure 3:**
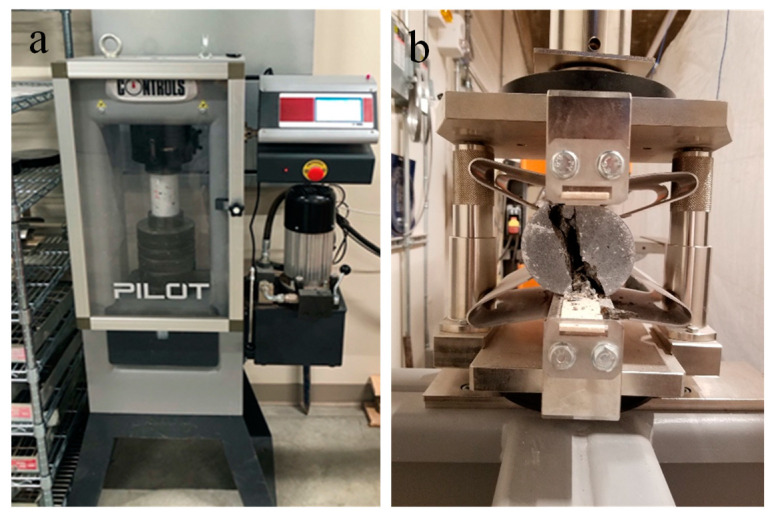
Setup for (**a**) compression, and (**b**) split tensile test.

According to ASTM C39 [83] and ASTM C109 [81], the compressive strength of concrete cylinders and mortar cubes was measured, respectively, with three repetitions, on a PILOT automatic compression tester at a uniform loading rate of 126 MPa/s (Figure 3a). The split tensile test was conducted according to ASTM C496 [84] at a loading rate of 83 MPa/s (Figure 3b) to measure the splitting tensile strength of the concrete cylinders with and without 5 vol.% capsules.

#### 2.3.4. TGA

TGA was performed to monitor the degradation behavior of the PLA capsules inside the concrete, mortar, cement paste under different curing conditions. The degraded PLA capsule shells were extracted using tweezers from the fracture surfaces of the specimens after compression and split tension tests. The collected capsule shell chips were cleaned by removing cement residues and were dried at 80 °C for 24 h to eliminate the free water. The TGA tests were carried out on a Perkin Elmer TGA4000 (Waltham, MA, USA) in a temperature range from 30 °C to 800 °C at a heating rate of 10 °C /min under N_2_ inert atmosphere (flow rate of 20 mL/min). The tangent method was employed for data analysis on the TGA and the derivate TG (DTG) curves

#### 2.3.5. Crack-Healing Capacity

**Figure 4:**
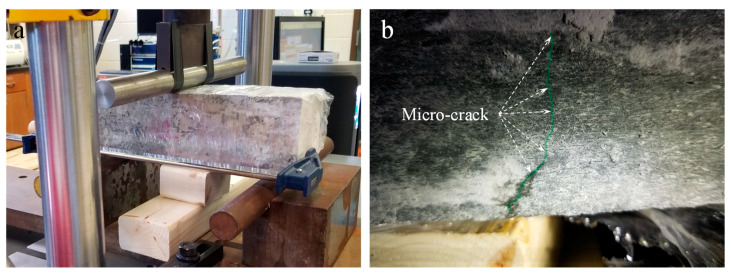
Self-healing test setup: (**a**) three-point loading; and (**b**) flexural crack formation.

A proof-of-concept test for the crack-healing capacity of concrete containing the developed biomass capsule system was studied by introducing flexural cracks in concrete beams after 90 days of curing. The release of sodium silicate from the capsules and crack closures were monitored. The concrete beams were loaded in a three-point bending setup by stages on an Instron universal testing machine as shown in Figure 4a. A bar clamp to introduce about 3500 N post-tensioning forces at the ends of the beam and wooden support in the middle were used to prevent brittle failure. The loading rate was reduced from 890 N/min to 445 N/min as the cracking load of the beams was approached. Immediately after the formation of flexural cracks (Figure 4b), the beam was unloaded and then cured in a saturated lime solution under room temperature (23 ± 2 °C) over the subsequent two weeks to monitor the healing of the cracks.

#### 2.3.6. Microstructure Analysis

The micromorphology of the aged gravel-level capsules in concrete after 90 days and sand-level capsules after being embedded in mortar for 270 days was performed on a digital microscope and a JSM-7401F field-emission scanning electron microscope (SEM) (JEOL USA, Peabody, MA, USA) under an accelerating voltage of 10 kV, respectively. The samples used for imaging were taken from the fracture surface of concrete and mortar specimens after the compression and split tension tests. For the SEM test, the sample surfaces were made conductive by sputtering deposition of a thin layer of gold using a Denton vacuum sputter coater.

## 3. Results and Discussion

### 3.1. Survivability of the Capsules in Concrete Mixing

The survivability of capsules during concrete mixing is characterized by determining the resistance of the capsules to breakage during regular concrete mixing. The capsules which did not break during mixing are denoted as intact capsules, among which the ones that did not leak after mixing are known as active capsules. Therefore, two ratios were determined: (i) intact ratio, which is the percentage of intact capsules, and (ii) survival ratio, which is defined as the ratio of the active capsules to the total number of capsules of each group. It should be noted that the survival ratio is equal to or lower than the intact ratio as leaking can be observed from some intact capsules. Previous research [85,86] has shown that the parameters that affect the capsule’s survivability include geometry, dimension and shell thickness, which were all investigated in this study.

#### 3.1.1. Influence of Capsule Size

**Figure 5:**
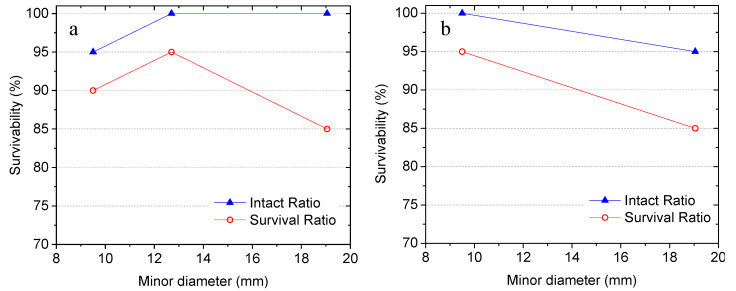
Influence of capsule size on survivability: (**a**) aspect ratio of 1.5:1:1, and (**b**) aspect ratio of 2:1:1.

Figure 5 shows the influence of the dimension of the capsules on their survivability at two different aspect ratios. The comparisons were made based on capsules, which have the same geometry and shell thickness but different sizes. From Figure 5a, it can be seen that, at an aspect ratio of 1.5:1:1 and a shell thickness of 0.8 mm (i.e. groups G2, G3, and G9 in Table 2), the capsule size did not exhibit a significant impact on the intact ratio of the capsules. G9 with a minor diameter of 9.5 mm yielded an intact ratio of 95%, and the other two groups with a minor diameter of 12.7 mm and 19.05 mm exhibited a higher intact ratio of 100%. However, comparing with the capsules with smaller sizes, a lower survival ratio (85%) was observed from the capsules with a minor diameter of 19.05 mm. This might be due to the fact that, with increasing volume, the possibility of printing defects during manufacturing and the probability of being impacted and abraded during concrete mixing were both increased. Figure 5b shows the comparisons between groups G4 and G7, where the capsules share the same aspect ratio of 2:1:1 and shell thickness of 0.8 mm. As the minor diameter of the capsules increased from 9.5 mm to 19.05 mm, the intact ratio and survival ratio decreased by 5% and 10%, respectively. It is worth noting that all of the five groups yield acceptable survivability with intact ratios higher than 95% and survival ratios over 85%. Nevertheless, capsules with smaller sizes are more preferred to survive the concrete mixing process with a lower possibility of leakage.

#### 3.1.2. Influence of Aspect Ratio

**Figure 6:**
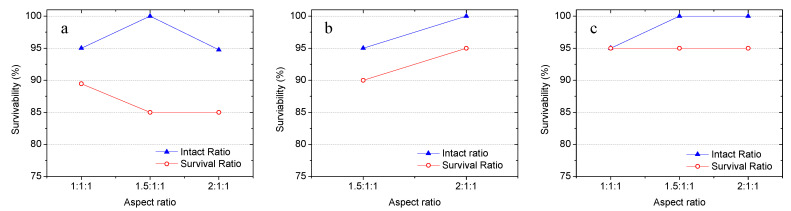
Influence of aspect ratio on the survivability of capsules with (**a**) a minor diameter of 19.05 mm and thickness of 0.8 mm, (**b**) minor diameter of 9.5 mm and thickness of 0.8 mm, and (**c**) minor diameter of 9.5 mm and thickness of 0.4 mm.

The influence of aspect ratio on the survivability of the capsules with two different minor diameters (i.e. 9.5 mm and 19.05 mm) is also investigated. As shown in Figure 6a, with a minor diameter of 19.05 mm and shell thickness of 0.8 mm, the capsules with an aspect ratio of 1.5:1:1 exhibited the best intact ratio for 100% while the capsules with lower (1:1:1) and higher (2:1:1) aspect ratios showed 5% lower capacity to maintain intact after concrete mixing. The highest survival ratio of 90% was obtained from the spherical capsules (1:1:1) and a 5% lower ratio was yielded by the two ellipsoidal groups. At a minor diameter of 9.5 mm, the elongated ellipsoidal capsules (2:1:1) showed better both intact and survival ratios than the capsules with an aspect ratio of 1.5:1:1 (see Figure 6b). As for 0.4 mm thickness, while the two groups of ellipsoidal capsules exhibited a higher intact ratio (100%) than the spherical capsules, the same survival ratio of 95% was observed from all of the three groups. Combining with the observations from Figure 5, capsules with a smaller size and higher aspect ratio is beneficial in achieving higher survivability. It worth noting that, compared with spherical capsules at the same volume fraction, the elongated ellipsoidal capsules will have a higher capacity to capture concrete cracks. The comparison between Figure 6b,c also indicates, at least in part, that the reduced shell thickness from 0.8 mm to 0.4 mm does not impact capsules’ survivability.

#### 3.1.3. Influence of Shell Thickness

**Figure 7:**
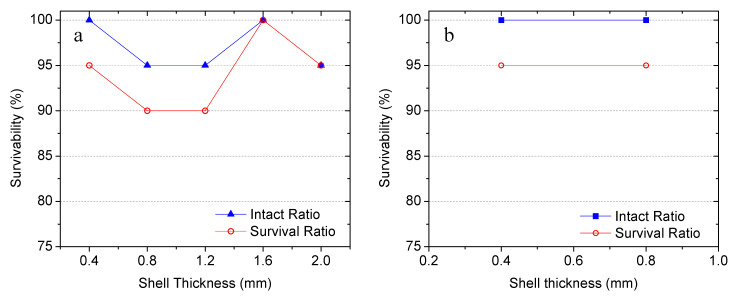
Influence of shell thickness on the survivability of capsules with aspect ratios of (**a**) 1.5:1:1, and (**b**) 2:1:1.

The influence of shell thickness on the survivability of capsules at the 9.5 mm (3/8 in.) gravel level was investigated for two aspect ratios (1.5:1:1 and 2:1:1). As shown in Figure 7a, at an aspect ratio of 1.5:1:1, the increase of shell thickness from 0.4 mm to 2.0 mm did not result in improved survivability. The capsules with a shell thickness of 0.4 mm yielded intact and survival ratios of 100% and 95%, respectively. The 0.8 mm and 1.2 mm shell thickness decreased both intact and survival ratios by 5%. Only the thickness of 1.6 mm yields a higher survival ratio than the 0.4 mm thick capsules. The same observations are obtained from the comparisons between groups G6 and G7, which have the same size and diameter-to-length ratio of 2:1:1. From Figure 7b, it can be seen that an increase of shell thickness from 0.4 mm to 0.8 mm resulted in the same intact ratio (100%) and survival ratio (95%) from the two groups. Furthermore, at the same volume fraction, the increased shell thickness can reduce the inner volume of the capsules thereby impacting the delivery efficiency of healing agents.

These results highlight the tremendous potential of the biomass capsules to survive the harsh concrete mixing condition and suggest promising strategies for self-healing concrete. Based on the systematic investigation and result analysis of the 12 groups, it can be concluded that, due to PLA’s excellent mechanical properties and the precise 3D printing process, all groups show acceptable survivability with the lowest survival ratio of 85%. To leverage this merit, and maximum the delivery capacity and the switchable properties of the biomass capsules triggered by alkaline degradation of PLA, the group exhibiting the highest survivability with a minor diameter of 9.5 mm, an aspect ratio of 2:1:1, and a shell thickness of 0.4 mm (G6) was selected for the following concrete-level investigations.

### 3.2. Degradation Behavior of Capsule Material

After addressing the challenge of survivability, the capsules need to be susceptible to concrete cracks to achieve efficient cracking capacity. Upon concrete cracking, the capsules should be able to capture the cracks and rupture to release core healing agents for crack healing. To achieve this goal, in this study, a concept of evolutionary property is proposed. Although the PLA-based biomass capsules are strong enough to survive the concrete mixing process, their mechanical properties would decrease in concrete triggered by the alkaline nature of the cement matrix and the degradation behavior of PLA. To validate this hypothesis, the degradation behavior of PLA and capsules in alkaline solution simulating concrete pore solutions and in the matrices of concrete, mortar and cement under different curing conditions were investigated.

#### 3.2.1. Degradation in An Alkaline Solution

**Figure 8:**
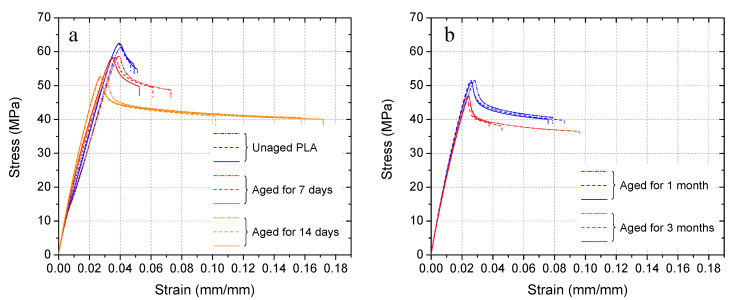
Stress and strain curves of PLA dog-bone specimens in tension test: (**a**) Raw PLA and aged PLA after alkaline treatment for 7 and 14 days, (**b**) aged PLA after alkaline treatment for 1 and 3 months.

The degradation rate of PLA in the 0.1 M sodium hydroxide (NaOH) solution with a pH of 13, which is similar to that of the pore solutions in concrete, was determined by monitoring the tensile strength loss of dog-bone samples over time. Figure 8a,b shows stress and strain curves of PLA dogbone specimens in the tension test. From Figure 8a, it can be seen that the raw PLA yielded the highest tensile strength. With 7 and 14 days’ alkaline solution treatment, the tensile strength was decreased while the slope of the linear stage was increased indicating that a higher Young modulus was yielded by these aged specimens. It was also observed that elongation at break was increased by alkaline treatment. As shown in Figure 8b, with longer emersion in the alkaline solution, the yield tensile strength was further decreased and a lower breaking strain was observed. The calculated tensile strength, strain capacity, and Young’s modulus are summarized in Table 3. The raw PLA (unaged) showed a brittle behavior with an average strength of 62 MPa, a modulus of 1.5 GPa, and an average rupture strain of less than 0.05 mm/mm. The maximum stress decreased with the increase of immersion time in alkaline solution. While an increased elongation at break was found from PLA after being immersed in the alkaline solution for 7-day and 14-day alkaline treatment, the strain capacity then decreased with further alkaline treatment for 1 month and 3 months. The tensile strength of PLA decreased by 6%, 15%, 17%, and 24% after the 7-day, 14-day, 1-month, and 3-month alkaline aging treatments, respectively. Achieving high survivability of capsules in concrete mixing is challenging, but synchronously attaining easy rupture upon cracks in the concrete matrix is more difficult and has rarely been achieved. It is believed that the reduced strength and increased brittleness of the aged PLA specimens in the alkaline solution indicate the potential of this material in achieving switchable properties of capsules in the alkaline environment of concrete.

#### 3.2.2. Degradation of Gravel-Level Capsules in the Matrix of Concrete

**Figure 9:**
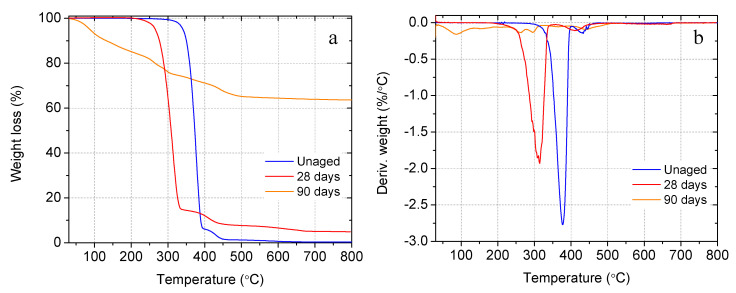
Comparison of unaged PLA and after degradation in concrete cured inside saturated lime solution at 23 °C for different ages: (**a**) thermogravimetric weight loss, and (**b**) derivative of weight loss.

Given the importance of switchable properties in controlling the release of healing agents and crack healing, the degradation behavior of PLA capsules in the matrices of concrete, mortar, and cement paste under different curing conditions was investigated via TGA on capsule samples extracted from fracture surfaces of cylinder and cube specimens after strength tests. Figure 9a,b shows the TGA and DTG curves of the raw and aged capsules, after being embedded in concrete specimens, which were cured in saturated lime solution at 23 °C, for 28, and 90 days, respectively. From the TGA and DTG curves, the initial degradation (onset) temperature and the maximum degradation temperature, i.e. the temperature for the highest thermal degradation rate, were calculated and summarized in Table 4. It can be seen that the raw PLA exhibited a two-stage combined weight loss of 97% between 356 °C up to 445 °C, while the capsule extracted from 28-day concrete showed a weight loss of 88.9% suggesting an 8.1% alkaline degradation. The first degradation step (~93% weight loss) between 356 °C and 390 °C is due to the breakage of the ester groups resulting in chain-scission of the PLA macromolecules into smaller fragments [87]. The second smaller weight drop (~4%) between 390 °C and 443 °C is due to the degradation of the smaller fragments formed in the previous degradation step [88]. After 90 days, it was found that at least 90.2% of PLA has been degraded in concrete. The multiple weight loss steps observed from the 90-day sample are due to the residual cement hydration products bonded on the capsule surface, which can be evidenced by the characteristic weight loss temperature ranges of C-S-H and portlandite. 

In addition to the reduced weight loss, decreased thermal stability was also observed from the aged PLA capsules. As shown in Table 4, after 28 and 90 days’ embeddedness in concrete, the onset degradation temperature of the capsules was decreased by 71 °C, and 108 °C, respectively, from the unaged specimens indicating that the structure of PLA may have been altered in the alkaline environment of concrete. The maximum degradation temperature was reduced by 9.7 °C and 140.2 °C, respectively, after 28 and 90 days’ degradation in concrete. The ash content indicates the amount of residue after heating the sample up to 800 °C. The raw PLA, with a final degradation temperature of about 443 °C, has less than 1% ash content signifying the entire thermal decomposition. As summarized in Table 4, the ash content increased up to 63% after 90 days. This is mainly due to the cement hydration products bonded to the degraded PLA capsule surface with a higher final degradation temperature than PLA.

#### 3.2.3. Degradation of Sand-Level Capsules in the Matrix of Mortar

**Figure 10:**
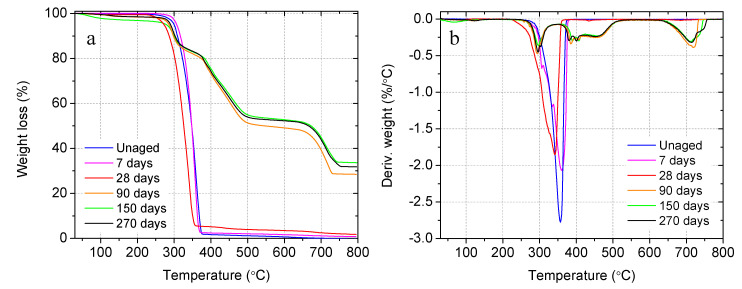
Degradation of PLA capsules in mortar cured in a saturated lime solution at 23 °C: (a) thermogravimetric weight loss, and (b) derivative of weight loss.

The TGA and DTG curves of the unaged and aged sand-level biomass capsules in the matrix of mortar cured in saturated lime solution at 23 °C are shown in Figure 10. As discussed above, these capsules were made of the transparent filament. It is worth noting that, different from the two-stage thermal decomposition of the white PLA filament in the previous TGA test, the transparent PLA exhibited a single-stage weight loss between 326 °C and 374 °C. This may be because the smaller fragments of PLA macromolecules, produced by the breaking of ester bonds between 326 °C and 374 °C, do not undergo any further degradation between 374 °C and 445 °C. As summarized in Table 4, compared with the raw PLA, the PLA capsules extracted from mortar specimens after 7 and 28 days of curing showed slight degradations of 0.1%, and 3.4%, respectively. The degradation increases over time with a maximum degradation of 86% after 150 days. An extended 270 days curing did not result in further capsule degradation. Similar to the capsules inside the concrete matrix, there is a decrease in the thermal stability manifested by a reduction of onset temperature by about 30 °C and 50 °C after being embedded in the mortar for 28 and 90 days, respectively. This is due to the change in the molecular structure and reduction of molecular weight of the PLA under alkaline degradation, which occurs via random scissions of ester linkages. The molecular weight decreased exponentially since this process is autocatalytic in nature [76]. In agreement with the extent of degradation, the maximum decrease in onset temperature was reached after 90 and 150 days and no further loss was observed from 270 days. The maximum degradation temperature was reduced by 23 °C and 64 °C after being embedded in mortar for 28, and 90 days, respectively, indicating the debinding of PLA macromolecules and a reduction of molecular weight in the alkaline environment of the mortar matrix. Limited ash content was observed from the capsule shells cured up to 28 days. However, beyond 28 days, the ash content increased over time to a high level of over 30%. Again, this might be due to the hydration products inside or bonded to the surface of the degraded PLA capsules. This can be evidenced by the TGA weight losses and DTG peaks in the temperature ranges of 370–520 °C and 640–750 °C, which are typical decomposition temperatures of portlandite and calcite, respectively.

#### 3.2.4. Degradation of Capsules in Cement Paste under Elevated Temperature

**Figure 11:**
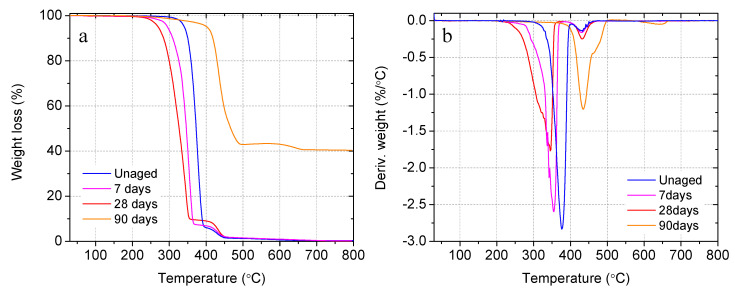
Comparison of degradation of PLA capsules in cement paste under 50 °C lime water: (**a**) thermogravimetric weight loss, and (**b**) derivative of weight loss.

In addition to the room temperature, the degradation behavior of the biomass capsules in the matrix of cement paste was also investigated under an elevated temperature of 50 °C. Figure 11a,b shows the TGA and DTG curves of raw PLA and the aged capsules extracted from the cement paste specimens. Similar to the previous observations from concrete and mortar, a slight degradation of 0.3% and 0.4% at 7 and 28 days, respectively, was observed. The degradation increases gradually up to 41.7% after 90 days as shown in Table 4. Again, two degradation steps were observed from both raw and aged capsule shells. The second weight loss increased progressively over time. This might be caused by multiple reasons: (i) the elevated curing temperature accelerated the breakage of the ester bonds of the macromolecules resulting in a large quantity of the smaller fragments, which were responsible for the decreased first weight loss and the increased second weight loss; (ii) mineralization of the capsule shell caused by the ingress and growth of cement hydration products dominated by portlandite inside PLA fibrils, which can also be promoted by the elevated temperature. Interestingly, the thermal decomposition of portlandite shares a similar temperature range with the second PLA degradation step. This mineralization phenomenon was observed in the microstructure analysis and will be discussed in Section 3.5. Further quantitative characterization is needed in future works to determine the contribution of cement hydration products in the second weight loss. Decreased thermal stability was also observed from the decreased onset temperatures by 29 °C, 55 °C and 103 °C after 7, 28 and 90 days, respectively, due to the gradual reduction of molecular weight in alkaline degradation. Due to overlapping of the final degradation temperature of the degraded PLA and portlandite attached to the PLA surface, no significant decrease in the maximum degradation temperature (460.7 °C) was observed even after 90 days. This again can be evidenced by the increased ash content at 800 °C. During the first 28 days, a small amount of ash content (<1%) was observed suggesting the nearly entire incineration of the PLA capsules. After 90 days, the ash content increased to 41.1% due to the residue of cement hydration products (portlandite).

The combined results from the capsules embedded in the matrices of concrete, mortar and cement paste reveal a slow degradation of PLA-based capsule shells during the first 28 days and a significant degradation up-to 90 days indicating the potential of this biomass capsule system to achieve good compatibility with the matrices to eliminate negative impact on workability and strength gain of young concrete and also ensure crack healing in mature concrete. Further investigations in these two aspects are shown in Section 3.3 and Section 3.4.

### 3.3. Influence on Mechanical Properties of Concrete and Mortar

To further understand the compatibility of the two biomass capsule systems in mortar and concrete, the development of the mechanical strength of concrete cylinders and mortar cubes was investigated. Although the degradation rate of the biomass capsules can be accelerated under elevated temperatures, to approximate the strength evolutions of concrete and mortar in real structures, this investigation was conducted on samples cured under room temperature.

#### 3.3.1. Influence of Gravel-Level Capsules on Concrete Strength

**Figure 12:**
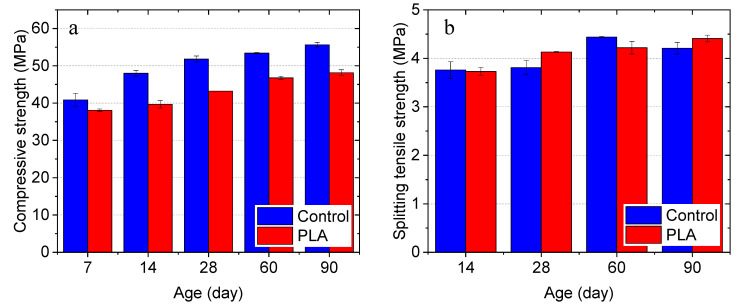
Strength development of concrete cylinders in (**a**) Compression, and (**b**) tension.

**Figure 13:**
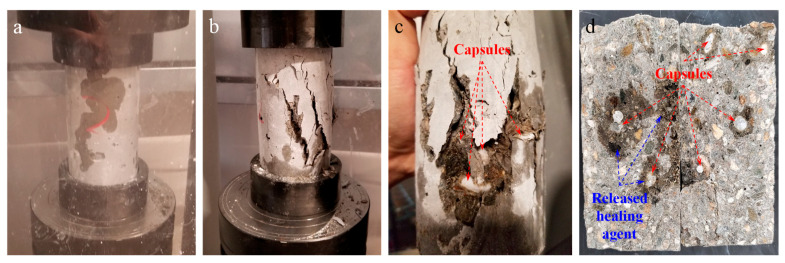
Exudation of self-healing agent: (**a**) and (**b**) during, and after (**c**) compression and (**d**) split tension test.

Influences of the gravel-sized capsules on the development of compressive and splitting tensile strength of concrete cylinders are shown in Figure 12a,b, respectively. From Figure 12a, it can be seen that, compared with plain concrete, the incorporation of 5% capsules by volume resulted in a decrease in 7-day compressive strength by 6.8%. Although the biomass capsules experienced degradation in the alkaline concrete matrix, compressive strength gain from 7 days to 90 days was still observed from the concrete containing capsules. However, the strength increasing rate is lower than that of the control group (plain concrete). After 28 and 90 days, the compressive strength of the capsule-containing concrete was 18.5% and 13.5%, respectively, lower than the plain concrete. A few possible causes have been considered for this phenomenon. First, this might be due to the degradation of the capsules inside the concrete to serve as possible pathways of crack propagation under loading, which tends to follow a path that provides the least resistance. As shown in Figure 13a, exudation of the liquid healing agent during the compression test was observed indicating that the rupture of capsules occurred due to internal crack propagation even before concrete failure. Second, in this study, although the capsules in gravel scale can guarantee healing agent delivery capacity of single capsules and the fraction of capsules broken by the crack, they serve as large inclusions in the matrix of concrete creating larger potential defects for stress concentration under loading. Two fracture patterns with diagonal fracture (Figure 13b) and columnar vertical cracking (Figure 13c) were observed from capsule-containing concrete cylinders. More importantly though, according to previous studies by Jung [89], Zhang [56] and Huang and Ye [90], there is no effect on strength when the volume fraction of capsules is at 2 %, while a 5% volume fraction can result in decreased strength and Young’s modulus of concrete.

As shown in Figure 12b, different from the compressive strength of concrete, the incorporation of capsules resulted in comparable and even increased splitting tensile strength. After 28 and 90 days, the capsule-containing concrete yielded splitting tensile strength of 4.1 MPa and 4.4 MPa, which were 8.4% and 4.8% higher than the plain concrete, respectively. Although concrete is not normally designed to resist direct tension, the results indicate that the resistance of concrete to cracking under transverse tension load was not negatively impacted by the biomass capsules. In addition, capsule ruptures and the induced healing agent release were also observed on the fracture surface of the concrete after the split tension test (see Figure 13d). 

#### 3.3.2. Influence of Sand-Level Capsules on Mortar Strength

**Figure 14:**
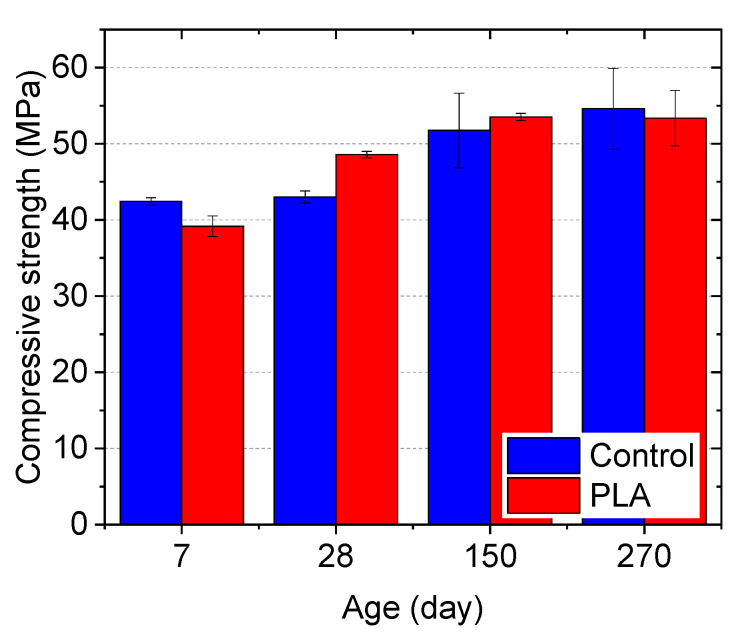
Comparison of compressive strength for mortar cubes with and without PLA capsules.

Comparing with the observations in concrete, the incorporation of sand-size capsules shows less impact on the compressive strength development of mortar. As shown in Figure 14, at 7 days, the capsule-containing mortar yielded a 7.7% lower strength than the plain mortar. However, the mortar containing capsules showed 13% and 3.4% higher strength than plain mortar after 28, and 150 days, respectively. After a long-term (270 days), the two groups exhibit comparable strength. This might owe to two reasons. On one hand, the smaller size of the sand-level capsules can generate fewer and smaller defects in the matrix than the gravel-level capsules. On the other hand, the lower volume fraction of capsules (2%) does not negatively impact composite strength, which is in agreement with the previous studies [89]. This observation indicates the acceptable compatibility of the sand-sized capsules in the matrix of mortar. 

### 3.4. Self-healing Capability

**Figure 15:**
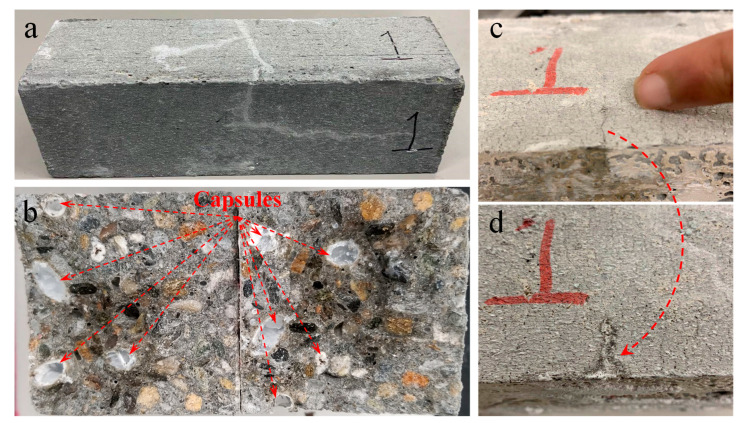
Morphology of (**a**) concrete beam surface, (**b**) fracture surface after bending, (**c**) and (**d**) crack closure.

This study focuses largely on the feasibility and compatibility of using the PLA-based biomass capsules in concrete. Therefore, in this study, the self-healing capacity of the capsule system was preliminarily investigated based on a fundamental proof-of-concept test. Evidence of inside-out seal-healing agent release via the newly formed cracks was observed on the surface of concrete cylinders during and after the compression and split tension tests (see Figure 13). From concrete beams before and after the flexure test, this phenomenon was further confirmed. As shown in Figure 15a, on the surface of concrete beam specimens, before the three-point bending test, dried sodium silicate from the capsules and its reaction products with the lime solution was found to cover the cracked area immediately after the crack formation. From the fracture surface of completely broken beams by flexural loading, as shown in Figure 15b, ruptured capsules and the distribution of the liquid sodium silicate were found. A 100% breakage ratio was yielded by the capsules distributed on concrete fracture surfaces. This again validates the switchable properties of the developed biomass capsules, which not only ensure the resistance against impact and abrasion during concrete mixing but also guarantee their easy rupture upon concrete cracks. From the beam specimens with successful micro-crack creation (Figure 15c), closure of a 0.5 mm wide crack was observed within a few hours (Figure 15d). The entire crack was filled with solid C-S-H gel formed by the reactions between sodium silicate and cement hydration products as well as the lime water. These observations indicate the potential of effective self-healing of concrete cracks triggered by the PLA-based biomass capsule system. However, additional investigations on crack self-healing efficiency in terms of internal crack repair, mechanical property recovery, permeability amelioration, and long-term aging resistance improvement still need to be performed in future works to obtain a comprehensive understanding.

### 3.5. Microstructure Analysis

**Figure 16:**
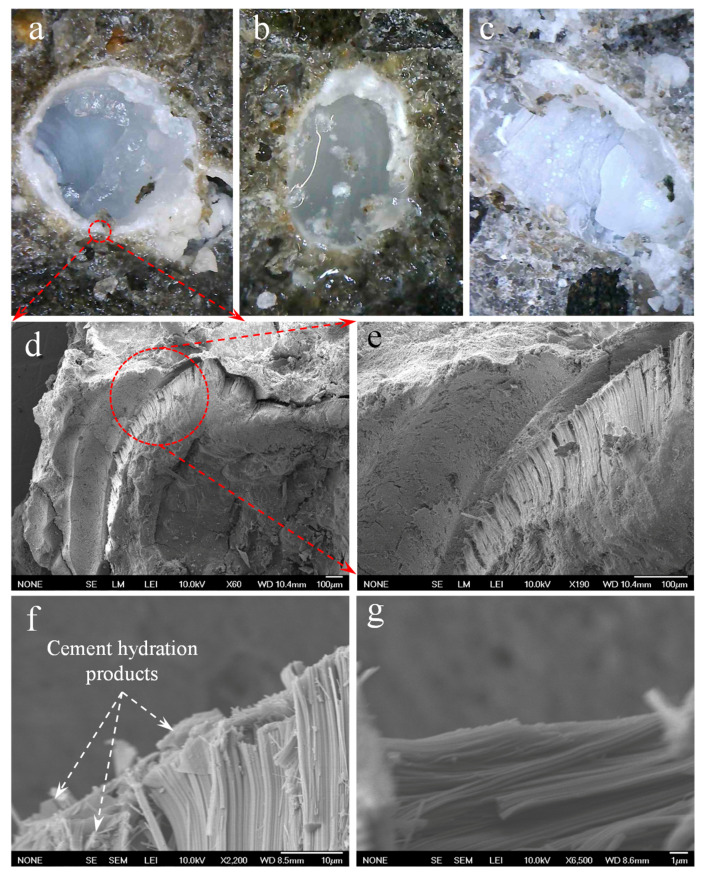
Capsules ruptured along (**a**) minor diameter and (**b**) major diameter containing liquid healing agent, (**c**) reaction products inside and around a ruptured capsule in concrete for 90 days under a digital microscope, (**d**) and (**e**) interface between a capsule and the cement matrix under SEM, (**f**) and (**g**) micro-texture of the ruptured biomass capsule in mortar after 270 days.

Micromorphology of the biomass capsules in concrete and mortar was characterized by a digital microscope and SEM under different magnifications. As shown in Figure 16a,b, after 90 days in concrete, depending on orientation, the ellipsoidal biomass capsules can be ruptured either transversely along their minor diameters or longitudinally along their major diameters. Again, this can be attributed to (i) weakening of the capsule shell due to degradation in the alkaline matrix of cement, (ii) suitable dimension and spacing of the capsules that can capture potential cracks, and (iii) good interfacial bonding between capsules and the matrix. The combination of these causes makes the cracks propagate through the capsules instead of bypassing them. As a result, the liquid healing agent inside the capsules can be effectively released in the presence of cracks. In the areas of capsule shells, gel-like products as a result of reactions between sodium silicate and the cement matrix can also be observed (see Figure 16a,b). Figure 16c shows the damaged capsules after 24 h of the rupture, from where more reaction products and hardened sodium silicate can be found. This agrees well with the observations from crack healing in Section 3.4. 

Under SEM, the interface between capsules and the cement matrix can be observed in more detail. From Figure 16d and its partial enlargement Figure 16e, the close contact of the capsule with the surrounding cement matrix can be observed from the fracture surface after compression test indicating the strong interfacial bonding between the two phases. In addition, micro-cracks were also observed from the outer layer of the degraded capsule shell. This is due to the stripping of microfibrils of PLA in the presence of an alkaline environment. These biomass microfibrils can experience both alkaline hydrolysis and mineralization in the matrix of cement [91]. From Figure 16f,g, both vertical and transversal stripped microfibrils, as well as cement hydration products distributed on the surface and inside capsule shells, can be observed. These microstructure observations explain the alkaline degradation behavior of the capsules in the matrix of cement as found from tension, TGA and rupture tests, and validate the great potential of the developed biomass capsule system in effective concrete self-healing.

## 4. Conclusions

The present study evaluates the feasibility and compatibility of using novel PLA-based biomass capsules as a potential carrier for the healing agents in self-healing concrete. Using the 3D printing technique, capsules with a variety of geometries and dimensions were manufactured. Survivability of the capsules during regular concrete mixing was determined based on intact and survival ratios. The effects of the gravel-size and sand-size capsules on the mechanical properties of concrete and mortar were evaluated. To validate the hypothesis of switchable properties of the biomass capsules in the matrix of cement, degradation behavior was monitored using a tension test, TGA, and microstructure analysis. A fundamental proof-of-concept test was also conducted to verify the crack self-healing efficiency of the concrete. The experimental results presented in this paper indicate the possibility of utilizing biomass components as property-switchable capsules to address the current challenges in self-healing concrete. The following conclusions can be drawn:

- Among the groups with a variety of dimensions and geopmetry shapes, elongated ellipsoidal capsules with a smaller size and higher aspect ratio yielded higher survivability, while shell thickness did not exhibit a discernible effect on the survival ratio of the biomass capsules. By considering survivability and healing agent delivery capacity (internal volume), capsules with a minor diameter of 9.5 mm, an aspect ratio of 2:2:1 and a shell thickness of 0.4 mm, which yielded intact and survival ratios of 100%, and 95%, respectively, were chosen for further studies on concrete properties.
- The switchable properties of the biomass capsules were validated by monitoring the alkaline degradation of capsule shell material. It was observed that, after immersing in an alkaline solution (pH 13) for three months, the tensile strength of PLA was decreased by 24.3%, while the Young’s modulus was increased by 26.6%. TGA results showed negligible degradation of PLA in the cementitious matrix during the first 28 days, whereas significant degradation was observed after 90 days under both 23 °C and 50 °C. Decreased thermal stability was also observed from the degraded capsule shells.
- Concrete containing a 5% volume fraction of gravel-sized biomass capsules showed a reduction in compressive strength of up to 18.5% compared to the plain concrete, while the splitting tensile strength was not negatively impacted. Incorporation of 2 vol.% sand-sized capsules resulted in comparable and even increased compressive strength of mortar.
- Based on a fundamental proof-of-concept test, the developed biomass capsule system showed promising self-healing prospects as a 0.5 mm wide flexure-induced crack introduced in a concrete beam was healed by solid C-S-H gel, which is formed as reaction products between sodium silicate healing agent, cement hydration products and lime water. The fracture surface of completely ruptured beams showed a 100% breakage ratio of capsules indicating successful release of self-healing agent into the surrounding cracks.
- The microstructure analysis showed that, due to the aged shells and good interfacial bonding with the cement matrix, the capsules can easily be raptured either transversely or longitudinally. Hardened silicate reaction products and sodium silicate can be observed around the raptured capsules. The mineralization of the capsule shell, indicated by the stripped-off micro-fibrils and embedded cement hydration products, was also observed.

## Figures and Tables

**Table 1:** Chemical composition of cement.

**Oxides Components (%)**	**SiO_2_**	**Al_2_O_3_**	**Fe_2_O_3_**	**CaO**	**MgO**	**SO_2_**		**CO_2_**
	19.9	4.8	3.1	62.2	3.4	3.6		0.5
**Phase Components (%)**	**C_3_S**	**C_2_S**	**C_3_A**	**C_4_AF**	**C_3_S + 4.75C_3_A**		**Equivalent Alkalis**	
	53	17	7	9	89		0.60	

**Table 2:** Summary of the capsule’s dimension and geometry for survivability test.

Group		X (mm)	Y (mm)	Z (mm)	Aspect Ratio	Shell Thickness (mm)
Sand level	S1	5	5	7.5	1.5	0.4
Gravel level	G1	19.05	19.05	19.05	1:1:1	0.8
	G2	19.05	19.05	28.575	1.5:1:1	0.8
	G3	12.7	12.7	19.05	1.5:1:1	0.8
	G4	19.05	19.05	38.1	2:1:1	0.8
	G5	9.5	9.5	9.5	1:1:1	0.4
	G6	9.5	9.5	19.05	2:1:1	0.4
	G7	9.5	9.5	19.05	2:1:1	0.8
	G8	9.5	9.5	14.25	1.5:1:1	0.4
	G9	9.5	9.5	14.25	1.5:1:1	0.8
	G10	9.5	9.5	14.25	1.5:1:1	1.2
	G11	9.5	9.5	14.25	1.5:1:1	1.6
	G12	9.5	9.5	14.25	1.5:1:1	2

**Table 3:** Summary of tensile test on dogbone PLA specimens.

PLA Specimen	Average Strain (mm/mm)	Yield Strength (MPa)	Break Stress (MPa)	Average Young’s Modulus (MPa)
Raw	0.0491	62.0	37.13	1523.11
7 Days Treatment	0.0622	58.43	33.12	1492.17
14 Days Treatment	0.1439	52.54	24.17	1514.27
1 Month Treatment	0.0803	51.32	24.05	1927.54
3 Months Treatment	0.0726	46.94	24.66	1928.32

**Table 4:** Degradation of PLA capsules in the matrices of concrete, mortar and cement paste under different curing conditions.

Matrix (Temperature)	Sample	Onset Temperature, T_d_ (°C)	Maximum Degradation Temperature (°C)	Weight Loss (%)	Degradation (%)	Ash Content at 800 °C (%)
-	Raw PLA^1^	356.0	445.0	97.0	-	0.4
	Raw PLA^2^	326.3	374.2	96.7	-	0.2
Concrete(23 °C)	28 days	284.6	435.3	88.9	8.1	5.0
	90 days	248.5	304.8	6.8	90.2	63.6
Mortar(23 °C)	7 days	330.7	365.4	96.6	0.1	0.8
	28 days	301.7	351.9	93.4	3.4	1.7
	90 days	280.3	307.8	13.0	83.7	28.5
	150 days	280.5	310.4	11.1	85.7	33.5
	270 days	282.7	310.6	11.8	84.9	31.8
Cement paste(50 °C)	7 days	327.1	444.4	96.7	0.3	0.2
	28 days	301.5	445.7	96.6	0.4	0.1
	90 days	253.5	460.7	56.2	40.8	41.1

PLA^1^: the white PLA used for capsules for survivability test and capsules embedded in concrete and cement paste; PLA^2^: the transparent PLA used for capsules embedded in mortar.

## Data Availability

The data presented in this study are available on request.
